# A systematic review of the prevalence of postamputation and chronic neuropathic pain associated with combat injury in military personnel

**DOI:** 10.1097/j.pain.0000000000003094

**Published:** 2023-12-15

**Authors:** Alexander Kumar, Nadia Soliman, Zoe Gan, Paul Cullinan, Jan Vollert, Andrew S.C. Rice, Harriet Kemp

**Affiliations:** a Department of Surgery and Cancer, Pain Research Group, Imperial College, London, United Kingdom; b Academic Department of Military Anaesthesia, Royal Centre for Defence Medicine, Birmingham, United Kingdom; c National Heart and Lung Institute, Imperial College, London, United Kingdom

**Keywords:** Chronic pain, Neuropathic pain, Phantom limb pain, Residual limb pain, Combat injury, Military

## Abstract

Combat trauma can lead to widespread tissue damage and limb loss. This may result in chronic neuropathic and post amputation pain, including phantom limb pain (PLP) and residual limb pain (RLP). The military population is distinct with respect to demographic, injury, and social characteristics compared with other amputation and trauma cohorts. We undertook a systematic review of studies of military personnel, with a history of combat injury, that reported a prevalence of any type of postamputation pain or chronic neuropathic pain, identified from Embase and MEDLINE databases.Using the inverse variance method with a random-effects model, we undertook a meta-analysis to determine an overall prevalence and performed exploratory analyses to identify the effect of the type of pain, conflict, and time since injury on prevalence. Pain definitions and types of pain measurement tools used in studies were recorded. Thirty-one studies (14,738 participants) were included. The pooled prevalence of PLP, RLP, and chronic neuropathic pain were 57% (95% CI: 46-68), 61% (95% CI: 50-71), and 26% (95% CI: 10-54), respectively. Between-study heterogeneity was high (I^2^: 94%-98%). Characterisation of duration, frequency, and impact of pain was limited. Factors reported by included studies as being associated with PLP included the presence of RLP and psychological comorbidity. The prevalence of postamputation pain and chronic neuropathic pain after combat trauma is high. We highlight inconsistency of case definitions and terminology for pain and the need for consensus in future research of traumatic injury.

## Abstract 2

Supplemental Digital Content is Available in the Text.

As a systematic review and meta-analysis of previously published data, no original data were collected for this study; therefore, it has not been made available.

## 1. Introduction

Combat polytrauma can result in significant mortality and morbidity because of the specific mechanisms of injury that occur in modern warfare. Although blast injury and penetrating trauma from projectiles are ubiquitous on the battlefield,^46^ terrorist attacks have meant that these injuries occur increasingly in civilian environments.^44^ Blast injury, from improvised explosive devices, became the signature injury of conflict in Afghanistan and Iraq over the past 20 years and, along with penetrating trauma from small arms fire, led to a characteristic pattern of wounding, including limb loss and widespread tissue damage.^65^ In earlier conflicts, similar catastrophic injuries were often lethal due to exsanguination on the battlefield or later from infection.^39^ Modern advances in battlefield medicine and trauma resuscitation have led to the emergence of a group of unexpected survivors who would have previously succumbed to their wounds but now live with the consequences of these injuries.^50^

The anatomical disruption caused by blast or penetrating injury can cause trauma to the nervous system^22^ and may give rise to neuropathic pain at almost any body location.^45^ Pain following amputation has been described in the context of military trauma for more than 500 years, with Ambroise Pare (1510-1590), a French military surgeon, identifying patients who reported pain in the missing limb after traumatic amputation.^37^ Over time, definitions of postamputation pain have changed as understanding of its mechanisms evolved: the most recent terminology is described in Table 1. The proposed mechanisms involved in phantom limb pain are diverse, including peripheral, spinal, and cortical changes and neuropathic symptoms are commonly reported. Injury to nerves at the level of the amputation can also give rise to neuropathic pain in the remaining part of the limb.^24,31,32,74^

**Table 1:** Current concepts and definitions of postamputation pain.

Residual limb pain	Spontaneous (continuous or paroxysmal) or evoked pain perceived as originating in the residual limb including the stump; pain unrelated to amputation, for example, other injuries, such as damage of the nerves above the level of amputation. The term residual limb pain can also *include* stump pain
Stump pain	Spontaneous (continuous or paroxysmal) or evoked pain in the amputation stump; includes neuroma, muscle, and bone stump as pain sources
Phantom limb pain	Spontaneous (continuous or paroxysmal) or evoked pain perceived as arising in the missing limb
Phantom limb sensation	Any sensation of the missing limb including pain

Modified from Edwards et al.^23^

It is helpful to differentiate neuropathic pain (pain arising as a direct consequence of a lesion or disease affecting the somatosensory system^35^) from nociceptive and nociplastic pain, as different underlying mechanisms necessitate different therapeutic strategies.^4^ Neuropathic pain associated with limb loss is particularly challenging to treat,^27,48^ has a significant impact on the quality of life, and can influence a patient's ability to engage with rehabilitation, resulting in an increased risk of long-term morbidity and disability.^26,52^ Despite this, the prevalence of chronic neuropathic pain associated with combat injury in military personnel is unclear.

Military personnel undertaking combat roles form a discreet population with a different socioeconomic and demographic profile compared with a civilian trauma population.^13,36,66^ At the time of injury, the military cohort are predominantly fit, healthy, young men operating in a specific psychosocial environment.^13^ Military veterans have specific health needs and have been recognised as a multifaceted group with a distinct culture that includes specific values, ethos, codes of conduct, and an obedience to command.^49^ They have also been identified as a group with greater use of opioid medication and a higher risk of overdose than the general population.^71^ Although recent systematic reviews of phantom limb^41^ and residual limb pain^43^ prevalence have determined prevalence in mixed civilian and military populations, encompassing diverse trauma and nontrauma aetiology, the prevalence and factors pertinent in a military setting and following solely traumatic aetiology are not known.

We undertook a systematic review of studies of combat injury to establish the prevalence of chronic neuropathic and postamputation pain following combat trauma. A secondary objective was to review the definitions used for chronic neuropathic pain and postamputation pain, and the types of assessment and tools used to explore pain in these conditions. Factors associated with the development of chronic neuropathic and postamputation pain were also explored. The review will also be used to inform and refine the “Chronic Neuropathic Pain After Combat Trauma” (CONTACT) study, which aims to prospectively establish the mechanistic classification, prevalence, and impact of pain within the Armed Services Trauma Rehabilitation Outcome Study (ADVANCE—https://www.advancestudydmrc.org.uk).^6^

## 2. Methods

The protocol was registered on PROSPERO (CRD 42020156892) on January 31, 2020, before the first formal literature search was conducted. Exploratory analyses of factors potentially influencing pain prevalence were performed in addition to analyses in the registered protocol. The review is reported in accordance with the Preferred Reporting Items for Systematic Reviews and Meta-analyses statement (PRISMA).^47^ The PRISMA checklist is included as Appendix 1, supplemental digital content, http://links.lww.com/PAIN/B946.

### 2.1. Literature search

The search strategy was constructed and informed using the “PICO” structure: population = military personnel or veterans; intervention = combat injury; comparator = none; outcome = postamputation pain (including phantom limb pain, residual limb pain, phantom limb sensation) prevalence or “other” neuropathic pain (not necessarily related to amputation) prevalence. Preliminary searches were undertaken to refine the search terms to assist in the appropriate construction of the search thread, which included Medical Subject Headings terms and free text. The search was performed using Embase and MEDLINE databases on the OVID platform. There were no restrictions on date or language. The first search was performed on May 10, 2021, and was updated on November 15, 2022 (For full list of search terms see Appendix 2, supplemental digital content, http://links.lww.com/PAIN/B946).

### 2.2. Study selection

Studies were exported to a reference management program (EndNote X9, Clarivate) where duplicate studies were removed. The studies were then uploaded to an online tool (Covidence; Covidence systematic review software, Veritas Health Innovation, Melbourne, Australia. Available at www.covidence.org). Title and abstract review, then full-text screening, was undertaken by 2 reviewers at each stage to confirm inclusion; a third reviewer was available to adjudicate disagreements.

All types of observational study were included, whereas case reports, opinion pieces, newsletters, comments, and editorials were excluded. Trials were included if information from the baseline characteristics was available and met inclusion criteria. Studies of civilian populations and those which included participants experiencing pain for fewer than 3 months after injury (therefore not fulfilling the definition of chronic pain^53^) were excluded.

The inclusion criteria used to define the population of interest were “military personnel” or “veterans” (defined as people who have served at least one day in the armed forces^72^). Combat injury was defined as an injury sustained whilst on active service during a military conflict. Where it was not clear if a described population included noncombat injury (eg, vehicular accident whilst in military training), a pragmatic cutoff of at least 70% explicitly described as combat injury was determined so as not to exclude useful data but to ensure that the majority of data referred to combat injury. For inclusion, studies were required to report prevalence data on chronic neuropathic pain and/or post amputation pain, including phantom limb and/or residual limb pain (including stump pain as per definitions in Table 1). The presence of postamputation or neuropathic pain reported by study participants, regardless of intensity or measurement tool, was used to define a subject with pain.

### 2.3. Data extraction and management

Data extracted from eligible studies were managed using Microsoft Excel. Initially, the template was piloted on 3 test papers to allow for refinement and development of an appropriate recording tool. The formal data extraction was performed by 2 independent reviewers (A.K. and Z.G. or H.K. and N.S.).

The primary outcome was to extract an overall prevalence for postamputation pain, including phantom limb syndrome (ICD-11 code: 8E43.00), phantom limb pain, residual limb pain (including stump pain), phantom limb sensation, and “other” chronic neuropathic pain (ICD-11 code: MG30.5). If stated, the type of prevalence was recorded (eg, point prevalence, period prevalence, lifetime prevalence). Study, participant, injury, and pain-related data were also extracted.

Study characteristics included year of publication, study design, study setting, data collection method, response rate, nationality of population, population size, and time since injury. Participant characteristics included age at study point, age at the time of injury, gender, ethnicity, relationship status, current work status, rank, serving personnel or veteran, and the conflict where the injury was sustained.

Injury characteristics of interest included the mechanism of injury (including blast injury [ICD-11 code: ND56.Y] or penetrating injury [ICD-11 code: ND56.1]), the New Injury Severity Score,^3^ traumatic amputation (ICD-11 code: ND56.8), delayed amputation, number of amputations, amputation site, other injuries, and the prevalence of mild traumatic brain injury (ICD-11 code: NA07.0).

Pain data included neuropathic pain definition used, postamputation pain definition used, diagnostic tool used, presence and location of other pain sites, pain assessment tools used, any factors associated with the development of chronic neuropathic or postamputation pain (including degree of association and the statistical method used to demonstrate association). Tools used to assess the quality of life and psychological comorbidities were collected, and the prevalence of posttraumatic stress disorder (PTSD) (ICD-11 code: 6B40), anxiety (ICD-11 code: MB24.3), and depression (ICD-11 code: 6A71) were recorded where available.

### 2.4. Risk of bias

The quality assessment checklist for prevalence studies by Hoy et al. was used to assess the risk of bias.^34^ This comprises 10 items and tests external and internal validity, giving a summary risk of bias assessment. Studies were considered low risk with a score of 0 to 3, medium risk if 4 to 6, and high risk with a score from 7 to 9. Risk of bias scores were not used as exclusion criteria.

### 2.5. Statistical analysis

A meta-analysis was performed for the prevalence of postamputation pain and “other” chronic neuropathic pain using the inverse variance method. Prevalences for phantom limb pain, residual limb pain, phantom limb sensation, and “other” chronic neuropathic pain were calculated using the random-effects model, and I^2^ was used to estimate the between-study heterogeneity. To prevent an overestimation of the precision of the prevalence, in the case of very low or very high proportions, we logit transformed the proportions before they were pooled to ensure normal sampling distribution and prevent biased standard errors.^42^ Prevalence was reported as percentage (95% confidence interval; calculated using the exact binomial method). We conducted stratified analysis by conflict and time since injury to investigate their influence on the prevalence estimate. Between-group heterogeneity was tested using the chi-squared distribution.

Any outliers (defined as having a confidence interval that does not overlap with the confidence interval of the pooled prevalence) were removed, and the analysis was repeated to assess the effect of the outlying result. Analyses were conducted on R version 4.0.3 and the package meta (version 6.1-0) and presented using forest plots.

Participant and study characteristics were summarised using median, interquartile range and range, or mean and standard deviation. Proportions of studies reporting pain definitions, pain characteristics, and comorbidities are presented as n (%). Factors identified to be associated with the presence of pain were described.

## 3. Results

**Figure 1.**
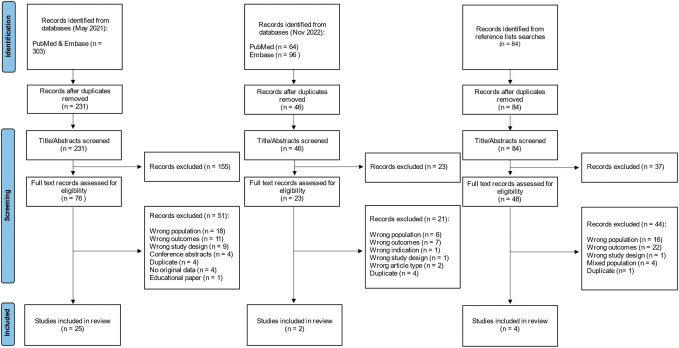
PRISMA flow diagram of study inclusion.

The original search, conducted in May 2021, returned 303 studies of which 72 were duplicates; 231 studies were screened by title and abstract and 155 studies did not meet the inclusion criteria and were removed. Full texts of 76 studies were assessed for eligibility with 51 studies excluded, leaving 25 studies to be included. An updated search was performed on November 15, 2022, where 46 were available for title/abstract screening after removing duplicates. Twenty-three were put forward for full-text screening, of which 2 met final inclusion criteria. Hand searching of references identified 84 studies to be screened, of which 4 were included in the final review. In total, 31 studies were included (Fig. 1). A list of excluded studies and reasons for exclusion are documented in Appendix 3 (supplemental digital content, available at http://links.lww.com/PAIN/B946).

### 3.1. Risk of bias

Table 2 illustrates how each study scored for internal and external validity using the Quality Assessment Checklist for Prevalence Studies.^34^ All studies scored a low risk of bias (5 studies scored 3 [16%]; 12 studies scored 2 [39%]; 8 studies scored 1 [26%]). The quality of internal validity was variable, with the main area of risk involving lack of definitions of key outcomes, including those used for different types of pain (lacking in 15 studies [48%]) and a lack of validated measures to assess pain (lacking in 22 (71%). Questionnaires were used in 25 studies with response rates quoted in 14, ranging from 26% to 100% (Table 3).

**Table 2:** Risk of Bias Assessment Based on the Quality Assessment Checklist for Prevalence Studies.^23^

Study	External validity				Internal validity					Score
	Was the study's target population (combat injured military personnel) a close representation of the overall military population in relation to relevant variables	Were those with pain after combat injury a true or close representation of combat injured personnel?	Was some form of random selection used to select the sample, OR, was a census undertaken?	Was the likelihood of nonresponse bias minimal?	Were data collected directly from the subjects (as opposed to a proxy)?	Was an acceptable case definition used in the study?	Was the study instrument used to measure pain after combat trauma shown to have reliability and validity?	Was the same mode of data collection used for all subjects?	Were the numerator and denominator for the parameter of interest appropriate	Summary on the overall risk of study bias
Aldington et al.^1^	0	0	0	0	0	0	0	0	0	0
Allami et al.^2^	0	0	0	0	0	1	0	0	0	1
Bedigrew et al.^5^	0	0	0	1	0	0	1	0	0	2
Birch et al.^8^	0	0	0	1	0	1	1	0	0	3
Buchheit et al.^10^	0	0	0	0	0	0	0	0	0	0
Duffy et al.^15^	0	0	0	0	0	0	0	0	0	0
Ebrahimzadeh et al.^18^	0	0	0	0	0	0	1	0	0	1
Ebrahimzadeh et al.^21^	0	0	0	1	0	0	1	0	0	2
Ebrahimzadeh et al.(a)^17^	0	0	0	0	0	0	1	0	0	1
Ebrahimzadeh et al.(b)^16^	0	0	0	0	0	0	1	0	0	1
Ebrahimzadeh et al.^19^	0	0	0	0	0	1	1	0	0	2
Ebrahimzadeh et al.^20^	0	0	0	0	0	1	1	0	0	2
Esfandiari et al.^25^	0	0	0	0	0	0	1	0	0	1
Faraji et al.^26^	0	0	0	0	0	1	1	0	0	2
Foote et al.^30^	0	0	0	0	0	1	1	0	0	2
Gunawardena et al.^33^	1	0	0	1	0	1	1	0	0	3
Ketz et al.^38^	0	0	0	0	0	0	0	0	0	0
Krueger et al.^40^	0	0	0	0	0	1	1	0	0	2
Rafferty et al.^51^	0	0	0	1	0	1	0	0	0	2
Rathore et al.^54^	0	0	0	1	0	1	1	0	0	3
Rauh et al.^55^	0	0	0	1	0	1	1	0	0	3
Rayegani et al.^56^	0	0	0	0	0	0	0	0	0	0
Reiber et al.^57^	0	0	0	0	0	1	1	0	0	2
Rivera et al.^58^	0	0	0	1	0	1	1	0	0	3
Rothberg et al.^59^	0	0	0	1	0	0	1	0	0	2
Sherman et al.^63^	0	0	0	0	0	1	1	0	0	2
Sherman et al.^64^	0	0	0	0	0	0	0	0	0	0
Sherman et al.^62^	0	0	0	0	0	0	1	0	0	1
Taghipour et al.^69^	0	0	0	0	0	0	1	0	0	1
Tintle et al.^70^	0	0	0	0	0	1	1	0	0	2
Wartan et al.^76^	0	0	0	1	0	0	0	0	0	1

1, No; 0, Yes.

Summary score: 0 to 3, low risk; 4 to 6, medium risk; 7 to 9, high risk.

**Table 3:** Characteristics of included studies.

Study	Nationality, publication y	Study design	Conflict	Cohort size, n	Study setting	Data collection	Response rate (%)
Aldington et al.^1^	UK, 2014	Cross sectional	Herrick/Telic 2001-2015	48	Amputees undergoing rehabilitation at DMRC Headley Court, UK	In-person survey	NR
Allami et al.^2^	Iran, 2019	Cross sectional	Iran–Iraq War 1980-88	247	Veterans listed by the Iranian Veterans and Martyrs Affairs Foundation in Tehran and Hamadan, Iran invited to participate in a health needs study	Interview	NR
Bedigrew et al.^5^	USA, 2017	Case controlled	OIF/OEF 2001-2015	38	ICD-9 codes used to search the US Department of Defence Trauma Registry	Medical record review	NA
Birch et al.^8^	UK, 2012	Cross sectional	Herrick/Telic 2001-2015	100	Patients attending the War Nerve Injury Clinic DMRC Headley Court, UK	Clinic assessment and medical record review	NA
Buchheit et al.^10^	USA, 2016	Cross sectional	OIF/OEF 2001-2015	124	Patients undergoing treatment at Walter Reed Military Medical Centre, USA	In-person questionnaire	NR
Duffy et al.^15^	Denmark, 2015	Retrospective review	Afghanistan war 2001-2015	53	Combat-injured Danish soldiers evacuated from Afghanistan to Denmark from 2006 to 2011	Self-administered questionnaires in the presence of investigator	65
Ebrahimzadeh et al.^18^	Iran, 2006	Cross sectional	Iran–Iraq War 1980-1988	25	War-related unilateral upper limb amputees in Veteran Administration of Khorasan Province, Iran	Interview/examination	NR
Ebrahimzadeh et al.^21^	Iran, 2007	Cross sectional	Iran–Iraq War 1980-1988	27	War-related foot/ankle amputees in Veteran Administration of Khorasan Province, Iran	Interview/examination	NR
Ebrahimzadeh et al.(a)^17^	Iran, 2009	Cross-sectional	Iran-Iraq War 1980-1988	31	War-related transfemoral amputees in Veteran Administration of Khorasan Province, Iran	Interview/examination	NR
Ebrahimzadeh et al.(b)^16^	Iran, 2009	Cross sectional	Iran–Iraq War 1980-1988	96	War-related unilateral transtibial amputees in Veteran Administration of Khorasan Province, Iran	Interview/examination	NR
Ebrahimzadeh et al.^19^	Iran, 2013	Cross sectional	Iran–Iraq War 1980-1988	76	Veterans from Iran–Iraq war with hip disarticulation or transpelvic amputation living in Iran	Interview/examination	90
Ebrahimzadeh et al.^20^	Iran, 2016	Cross sectional	Iran–Iraq War 1980-1988	291	Veterans with bilateral lower limb amputation registered with the Organisation of Veterans and Martyrs Affairs of Iran	Interview/examination	58
Esfandiari et al.^25^	Iran, 2018	Cross sectional	Iran–Iraq War 1980-1988	587	War amputees identified from the Veterans and Martyrs Affairs Foundation with knee disarticulation, transfemoral amputation, or hip disarticulation	Interview/examination	58.7
Faraji et al.^26^	Iran, 2018	Cross sectional	Iran–Iraq War 1980-1988	100	War amputees identified from the Veterans and Martyrs Affairs Foundation database	Interview/examination	48.8
Foote et al.^30^	USA, 2015	Mixed-method, Cross sectional	Vietnam War 1955-1975	247	Vietnam Veteran amputees recruited from the National Veteran Service Organisation, USA	Postal survey	59
Gunawardena et al.^33^	Sri Lanka, 2016	Case–control (amputee “case” group included)	Not stated	461	Servicemen or retired Sri Lankan war injured from 2 districts: Anuradapura and Kurunegala with unilateral lower limb amputation	Interview-administered questionnaire	98.3
Ketz et al.^38^	USA, 2008	Convenience sample	OIF/OEF 2001-2007	30	Military traumatic amputees attending an outpatient clinic at the Brooke Army Medical Centre, USA	Self-administered questionnaire	NR
Krueger et al.^40^	USA, 2015	Case series	OIF/OEF 2001-2015	44	Database of all late major extremity amputations at the Extremity Trauma and Amputation Center of Excellence Fort Sam Houston Texas, USA (2001-2011)	Medical record review	NA
Rafferty et al.^51^	UK, 2015	Cross sectional	Herrick/Telic 2001-2015	75	Military inpatients at DMRC Headley Court with amputation secondary to traumatic injury, UK	In-person questionnaire	100
Rathore et al.^54^	Pakistan, 2016	Cross sectional	War against terror Dates/location not defined	123	Consecutive patients reporting to the Armed Forces Institute of Rehabilitation Medicine (AFIRM), Pakistan with traumatic limb amputation (2007-2010)	Prospective survey and medical record interrogation	NR
Rauh et al.^55^	USA, 2013	Cross sectional	OIF/OEF 2001-2015	546	US Service members that underwent combat-related amputation whilst deployed on OIF/OEF	Medical record review	NA
Rayegani et al.^56^	Iran, 2010	Cross sectional	Iran–Iraq War 1980-1988	335	Veterans with bilateral lower limb amputations assessed at the Janbazan Medical and Engineering Research Centre, Iran	Interview	84
Reiber et al.^57^	USA, 2010	Cross sectional	OIF/OEF 2001-2015, Vietnam War 1955-1975	298 Vietnam 283 OIF/OEF	Vietnam veterans with limb loss identified through the VA Compensation and Pension Mini Master files OIF/OEF cohort with traumatic limb loss identified from the Madigan Army Medical Center M-2 Database and 2 VA databases, USA	Postal survey	65
Rivera et al.^58^	USA, 2014	Cross sectional	OIF/OEF 2001-2015	70	Medically discharged soldiers with combat injury at San Antonio Military Medical Centre Texas, USA	Medical record review	NA
Rothberg et al.^59^	USA, 1983	Epidemiological review	Vietnam War 1955-1975	7138	Data derived from the Patient Administration System of the US Army Surgeon General	Medical record review	NA
Sherman et al.^63^	USA, 1983	Cross sectional	Multiple	764	All members of the National Amputation Federation, USA	Postal survey	61
Sherman et al.^64^	USA, 1984	Cross sectional	Multiple	2694	5000 randomly selected veterans from a US Veterans Agency database of 25,000 known military-related amputees	Postal survey	55
Sherman et al.^62^	USA, 1999	Cross sectional	Multiple	45	US veterans with traumatic amputations over preceding 10 y, identified with the VA	Postal survey	26
Taghipour et al.^69^	Iran, 2009	Cross sectional	Iran-Iraq war 1980-88	141	Isolated unilateral lower limb amputees assessed at the Kowsar Prosthesis Centre for veterans, Iran	Interview with general physician	NR
Tintle et al.^70^	USA, 2012	Consecutive series	OIF/OEF 2001-2015	96	Medical record review of upper limb amputees from OEF/OIF performed at Walter Reed Military Medical Centre, USA	Medical record review	NA
Wartan et al.^76^	UK, 1997	Cross sectional	First and Second World Wars, Falklands War	526	Random selection of members from British Limbless Ex-Servicemen's Association database	Postal survey	89

NR, not recorded; NA, not applicable; OIF, Operation Iraqi Freedom—US military operations in Iraq 2003 to 2011; OEF, Operation Enduring Freedom—US military operations in Afghanistan 2001 to 2021; VA, Veteran's; DMRC, Defence Medical Research Centre; HERRICK, Operation HERRICK—British military operations in Afghanistan from 2002 to 2014; TELIC, Operation TELIC—British military operations in Iraq 2003 to 2011.

### 3.2. Study characteristics

Table 3 outlines the study characteristics of included studies. Overall, 14,738 participants from 6 national militaries were included. The largest cohort was of 7138 US Army participants with nerve injury,^59^ whereas the smallest study included 25 participants who had sustained unilateral upper limb amputations.^18^ Participants were injured in several significant conflicts; Afghanistan and Iraq (2001-2015), the Iran–Iraq War (1980-88), the Vietnam War (1955-1975), and one study including veterans from the Falklands War (1982) and 2 World Wars (1914-1918 and 1939-1945). One study was conducted in Sri Lanka but did not report a specific conflict^33^

Twelve studies, including 8 from the United States, 3 from the United Kingdom, and 1 from Denmark, reported pain outcomes after combat injury from Afghanistan and Iraq (2001-2015). One study from Pakistan reported pain in amputees from “the war against terror,” and although it did not specifically state the dates and location of the conflict, it is assumed to include injuries sustained in the preceding 2 decades related to the conflict in Afghanistan and Iraq.^54^ For the purposes of this review, the recent wars in Afghanistan and Iraq have been grouped together because in some of the literature, the participants are not clearly differentiated between the 2 conflicts.

Participants injured during the Iran–Iraq war were assessed in 11 studies, using a similar array of outcome measures to describe Iranian combat injured soldiers who were classified by the anatomical location of their amputation. Soldiers injured during the Vietnam War were represented in 3 studies, and 4 studies contained data relating to those injured in a variety of conflicts with conflict dates preceding 2000.

### 3.3. Participant and injury characteristics

The median (IQR, [range]) age of participants at the time of inclusion to the studies was 30.20 years (18.10 [18.00-89.00]), and 98.2% (95% CI: 97.0%-99.5%) were male. Nineteen studies stated whether the participants were veterans or still in active service at the time of the study, with 873 soldiers explicitly described as still serving, and 3381 reported as veterans at the time of assessment. The median time at assessment since injury was 17.40 (1.57-26.00 [0.37-50.00]) years. More than two-thirds, 68.5% (95% CI: 61.1%-76.0%) of injuries were due to blast and a further 30.8% (95% CI: 20.5-41.1) due to penetrating trauma. Three studies included data on traumatic brain injury with a mean of 23.5% (95% CI: 7.4%-39.6%) of respondents reported as having traumatic brain injury concurrently to other combat trauma (supplemental digital content, available at http://links.lww.com/PAIN/B946).

### 3.4. Pain prevalence

**Figure 2.**
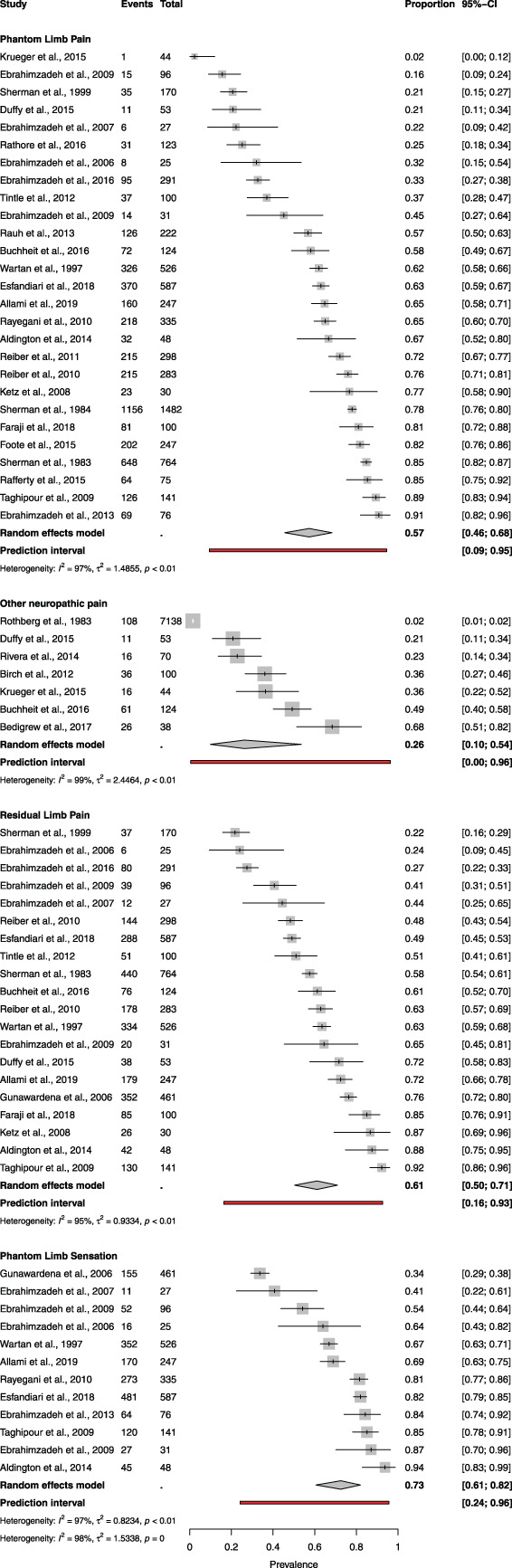
Forest plots of the prevalence pain in included studies, subgrouped by the type of pain. The size of the square represents the weight (%) that the individual subgroup has on the pooled result. “Events” denotes the number of participants with the pain condition among the “total” number of participants in the study.

The prevalences of phantom limb pain, phantom limb sensation, and residual limb pain were 57% (95% CI: 46%-68%), 73% (95% CI: 61%-82%), and 61% (95% CI: 50%-71%), respectively. The prevalence of “other” chronic neuropathic pain was 26% (95% CI: 10%-54%) (Fig. 2). Between-study heterogeneity within each type of pain was also high (I^2^: 94%-98%), and a test for subgroup differences (χ^2^ = 10.07, df = 3 [*P* = 0.02]) suggests that pain type accounts for a significant proportion of heterogeneity.

#### 3.4.1. Phantom limb pain

Phantom limb pain prevalence was quoted in 27 studies with a range from 2% to 91% and a pooled prevalence of 57% (95% CI: 46%-68%). A descriptor or definition of phantom limb pain was only used explicitly in 10 studies^1,10,17,18,21,25,38,56,69^ and with varying descriptors, including “painful sensations felt in the part of the limb that had been removed” or “painful sensations in the missing limb”. Only 1 study specified the period prevalence of phantom limb pain (evaluated over a predefined period) as opposed to point or an undefined prevalence.^1^ No studies reported a prevalence for the term “phantom limb syndrome”.

An evaluation of phantom limb pain severity was described in 9 studies but was inconsistently reported. Aldington et al.^1^ used a “none, mild, moderate, severe” categorical verbal descriptor scale with 56% reporting symptoms of at least moderate severity. Other tools used included numerical rating scales (NRS)^10,38,63,64,76^ and visual analogue scales (VAS).^51^

#### 3.4.2. Residual limb pain

Residual limb pain prevalence was reported in 20 studies with a range of 22% to 92% and a pooled prevalence of 61% (95% CI: 50%-71%). The condition was defined in 12 studies using the following descriptors: “pain in the stump”,^1,2,10,20,64,76^ “pain in the remaining part of the limb”,^16,18,75^ “spasm of the stump,”^19^ and “painful sensations felt at the site of amputation.”^38^ Methods used to assess the severity of residual limb pain included verbal descriptors and NRS. Aldington et al. reported that 63% of their cohort were experiencing moderate to severe residual limb pain, using a NRS (0-10),^1^ Allami et al. quoted residual limb pain severity of 7.9 of 10 at worst,^2^ whereas Ebrahimzadeh et al. (2013)^19^ reported that 96.4% of participants described severe and unremitting residual limb pain. Buchheit et al. used the self-administered Leeds Neuropathic Symptoms and Signs assessment (S-LANSS)^10^ to assess the type of residual limb pain, with 59% fulfilling the criteria for neuropathic pain (S-LANSS score greater than 12). Only 1 study reported period prevalence with the remainder reporting point prevalence or undefined prevalence.

#### 3.4.3. Other chronic neuropathic pain

Other chronic neuropathic pain associated with combat injury was reported in 7 studies with a pooled prevalence of 26% (95% CI: 10%-54%). If Rothberg et al. is considered an outlier (defined as having a confidence interval that does not overlap with the confidence interval of the pooled prevalence), the pooled prevalence increased to 38% (95% CI: 25%-53%). No formal diagnostic criteria for neuropathic pain were reported in 5 studies, although 2 mentioned ICD codes relating to causalgia or neuropathic pain.^5,59^ The S-LANSS tool with the validated cutoff of greater than 12^7^ and the PainDETECT questionnaire^31^ were used in 1 study each.^10,15^

### 3.5. Exploratory analysis of factors influencing pain prevalence

**Figure 3.**
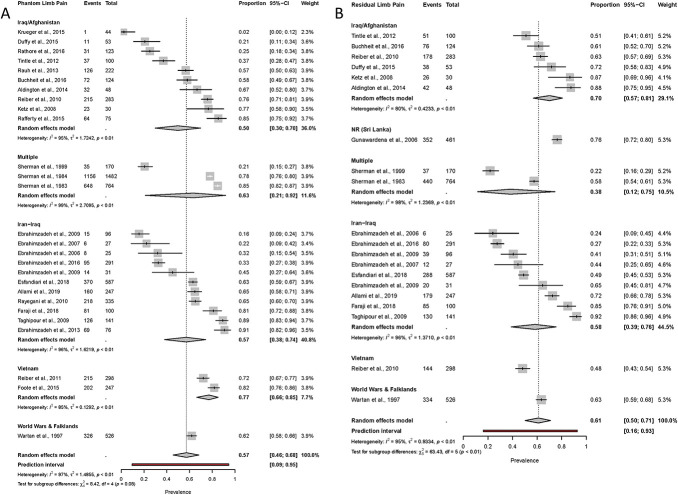
Forest plots of pain prevalence using the random-effects model for phantom limb pain (A) and residual limb pain (B) grouped by conflict. The size of the square represents the weight (%) that the individual subgroup has on the pooled result. “Events” denotes the number of participants with the pain condition among the “total” number of participants in the study.

Separate analyses were performed for phantom limb pain and residual limb pain because the type of pain was identified as a source of heterogeneity (Fig. 3). Analyses for neuropathic pain and phantom limb sensation were not performed due to the limited number of studies reporting this outcome measure. Prevalence for phantom limb pain (PLP) and residual limb pain (RLP) in the most frequently reported cohorts were from the recent Iraq/Afghanistan conflict: PLP = 50% (95% CI: 30%-70%) and RLP = 70% (95% CI: 57%-81%) and from the Iran–Iraq war: PLP = 57% (95% CI: 38%-74%) and RLP = 58% (95% CI: 39%-76%). Conflict accounted for a significant amount of heterogeneity for residual limb pain but not for phantom limb pain (PLP χ^2^ = 8.42, df = 4 [*P =* 0.08]; RLP= χ^2^ = 63.43, df = 5 [*P* < 0.01]).

**Figure 4.**
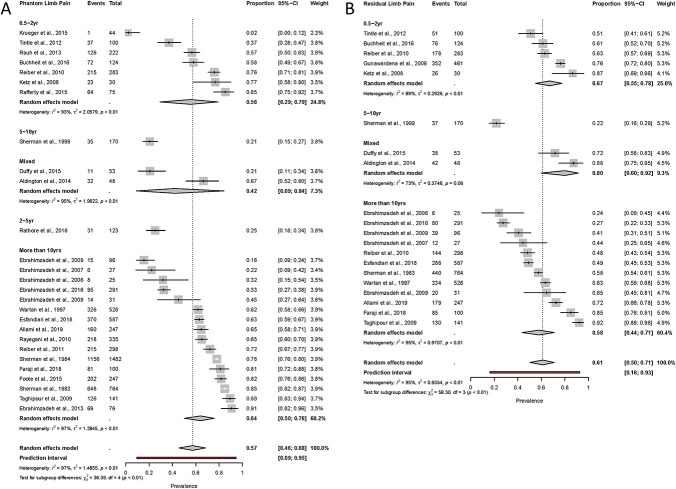
Forest plots of pain prevalence using the random-effects model for phantom limb pain (A) and residual limb pain (B) grouped by time since injury period. The size of the square represents the weight (%) that the individual subgroup has on the pooled result. “Events” denotes the number of participants with the pain condition among the “total” number of participants in the study.

Further analyses to identify the prevalence of phantom limb and residual limb pain by time since injury were also conducted (Fig. 4). Prevalence of phantom limb pain was 56% (95% CI: 29%-79%) and of residual limb pain was 67% (95% CI: 55%-78%) in studies reporting those injured most recently (6 months−2 years post injury). Prevalence of phantom limb pain was 64% (95% CI: 50%-76%) and residual limb pain was 58% (44%-71%) in the studies reporting injury more than 10 years since injury. Tests between subgroups for both phantom limb pain and residual limb pain suggest that the time since injury accounts for a significant amount of heterogeneity in both types of pain (PLP χ^2^ =36.39, df = 4 [*P* < 0.01]; RLP χ^2^ =58.30, df = 3 [*P* < 0.01]).

### 3.6. Factors with a reported association with postamputation or chronic neuropathic pain

Eight studies explored factors associated with the presence of postamputation and neuropathic pain.^2,10,15,16,63,64,70,76^ Across all studies, factors tested included participant characteristics (age, presence of comorbid pain diagnoses); psychosocial characteristics (measures of depression, anxiety, PTSD, pain catastrophizing, quality of life, relationship with other amputees); injury characteristics (level of amputation, presence of pain preceding amputation, time since amputation); and rehabilitation characteristics (prostheses use, residual limb complications). However, there was limited reporting of psychological and quality of life measures (supplemental digital content, available at http://links.lww.com/PAIN/B946). Phantom limb pain was associated with the presence of residual limb pain^64^ and phantom limb sensation,^76^ more distal amputation in upper limb amputation,^73^ and the presence of depression, anxiety, and PTSD.^17^ Residual limb pain was associated with higher intensity low back pain.^1^ Finally, neuropathic pain was associated with higher questionnaire-based scores for pain catastrophizing, depression, anxiety and PTSD, and lower quality of life.^10,15^

## 4. Discussion

To our knowledge, this is the first systematic review to quantify the prevalence of chronic neuropathic pain and postamputation pain (including phantom limb pain and residual limb pain) after combat trauma in military personnel. We identified 31 studies representing participants serving in a diverse set of conflicts and from a broad range of national armed forces.

One of the ongoing challenges in studying phantom limb pain is the heterogeneity in the reason for amputation. Amputation may occur because of a variety of trauma, infection, cancer, or vascular disease and, depending on the inciting event, occurs in a diverse range of age groups and demographic backgrounds. It is hypothesised that such differences may influence the risk of developing post amputation pain.^66,67^ However, our review is distinct from other recent reviews, which have included multiple aetiologies,^41^ as it focusses on a more homogeneous set of aetiologies and populations. First, this allows for comparison to identify if particular aetiologies, or populations, are at differential risk, but it also allows for the exploration of factors that are particularly relevant to combat trauma such as the specific conflict during which the injury was sustained.

The estimated prevalence of phantom limb pain from our analysis (57% [95% CI: 46%-68%]) is similar to that reported in a recent systematic review,^41^ which assessed the prevalence of phantom limb pain in participants from civilian and/or military backgrounds, with a range of amputation aetiology (39 studies; n = 12,738). It demonstrated a pooled prevalence estimate of phantom limb pain after limb loss of 64% (95% CI: 60%-68%). Although our analysis included a different cohort, reporting solely amputation related to combat trauma amongst military personnel, the similarity in prevalence could suggest that context and aetiology may be less influential than hypothesised.

Phantom limb pain has been described as decreasing in severity over time since amputation,^9,14,41^ but most studies in this area are cross sectional in design. The studies included in our review evaluated participants at a wide range of time since injury, from 13 weeks to 50 years. It should also be noted that all but one of the included studies describe either point prevalence or an unclear reporting time frame, rather than a defined period. Our exploratory subgroup analysis of effect of time on the prevalence of phantom limb pain indicated that prevalence may be influenced by time since injury. It should be emphasised that this was an exploratory analysis aimed at generating hypotheses and that only longitudinal studies will be able to provide a robust understanding of how time since injury influences the prevalence of pain. A higher prevalence in studies with a longer duration since injury may be because of an increased awareness and surveillance over time, where participants are asked more frequently about pain or are more aware of a condition that has received more attention in intervening years. There may also be sampling bias as those without pain are less engaged with the healthcare settings coordinating research and therefore are at a higher risk of being excluded.

Residual limb pain is a term used to describe a potentially heterogeneous set of underlying pain mechanisms and aetiologies.^11^ Pain in the residual limb can be attributed to specific pathologies such as neuroma or scar pain, but it may also encompass complex regional pain syndrome or nonspecific musculoskeletal pain. A systematic review by List et al.^43^ focussing on residual limb pain prevalence demonstrated a pooled prevalence of 59% (95% CI: 51%-67%), again with high heterogeneity (24 studies; n = 6716), which is similar to our calculated prevalence of 61% (95% CI: 50%-71%). Only 5 (out of 20) studies from the review by List et al. were included in our analysis as their review included all causes of amputation, suggesting that the prevalence may be similar in solely military settings. It is important to capture the diagnosis of residual limb pain when studying phantom limb pain because the presence of residual limb pain is the most commonly reported associated factor with the development of phantom limb pain.^41,66^ It also has the potential to impact on prosthesis use and, in some studies, has been demonstrated to be more problematic than phantom limb pain.^24^ In our review, we identified that very few studies linked the presence of residual limb to specific pain, function, or impact measures; therefore, the impact of this condition is currently unclear.

Our exploratory analyses suggest that a longer time since injury is associated with a slightly lower prevalence of residual limb pain and that the most recent conflict is associated with a higher prevalence. However, the differences in prevalence between the groups is small and with overlapping confidence intervals.

However, our exploratory analyses do not account for a large proportion of the considerable heterogeneity. Prevalence of phantom limb pain ranged from 2%^40,59^ to approximately 90%.^1,19,69^ The population in the study of Krueger et al.^40^ reporting a lower prevalence underwent relatively late amputation several months following initial injury, which may have influenced the development of long-term pain. The other study reporting low prevalence, that of Rothberg et al.,^59^ was based on medical record review rather than direct participant interview or questionnaire, and the presence of “causalgia” using ICD-8 coding was used as the primary outcome. Some studies have highlighted a lower prevalence of pain when assessed by clinician, as opposed to patient report,^68,75^ which may account for the particularly low prevalence. At the other extreme, Aldington et al.^1^ specified the time frame of “the previous 1 month” over which respondents were asked to recall pain. This may have led to a higher prevalence than studies which likely reported point prevalence rather than period prevalence. We hypothesise that although there are differences in injury characteristics, which may account for differences in prevalence, variety in data collection methods and definitions of painful conditions, which were commonly poorly reported, account for a greater amount of the observed heterogeneity.

### 4.1. Pain terminology

Our review highlights the challenges in interpreting pain prevalence data because of significant variability in the definitions and evaluation tools used for both chronic neuropathic pain and postamputation pain. The recent development of ICD-11 provides a refined classification of pain to include specific chronic pain aetiologies and includes codes for “chronic pain after amputation” in the “chronic postsurgical pain” category (MG30.21) and a category for chronic posttraumatic pain (MG30.20). Chronic pain after peripheral nerve injury has a separate code (MG30.51).^67^ Phantom limb syndrome (8E43.00) has been defined as “the perception of sensations, including pain, in a limb that has been amputated or a body part that has been removed. These sensations may include a specific position, shape, or movement of the phantom, feelings of warmth or cold, itching, tingling, or electric sensations, and other paraesthesias,” thereby including both painful and nonpainful elements. The update by Edwards et al. of the definitions for postamputation symptoms also provide a common lexicon which, if adhered to alongside the ICD-11 classification system, may reduce the uncertainty in analysing and comparing cohorts reporting pain following amputation.^23^

The definition and diagnostic criteria of neuropathic pain are also important because the condition has specific treatment and management pathways. Validated screening tools, such as the S-LANSS^7^ and PainDETECT,^31^ if used more consistently could provide a more detailed understanding of heterogeneity in pain mechanisms. The Neuropathic Pain Special Interest Group of the International Association for the Study of Pain has created a grading algorithm for the certainty of diagnosis of neuropathic pain.^28^ Only 2 studies in our review used validated tools and described cutoff scores for a positive case definition of neuropathic pain. The omission of this information leads to a high level of uncertainty about whether described “cases” of neuropathic pain could be considered comparable across the individual cohorts.

Across the studies, very few data describing the various dimensions of the pain experience were available. Differences in such qualities, such as intensity, duration, frequency, characteristics, and interference with physical and mental function, are key to understanding the impact of pain on the individual, to understanding heterogeneous pain-generating mechanisms, and to directing personalised management strategies. This lack of detailed information means that the prevalences reported likely include a range of pain intensity. Commonly, pain of “at least moderate” intensity is thought to be “impactful”; therefore, the prevalence of impactful pain cannot be determined from the current literature and may be significantly lower than the prevalences reported in this review. More information is needed in military cohorts to start to address the unique needs of those injured during combat.

### 4.2. Limitations

Although we have quoted a weighted prevalence for neuropathic pain and post amputation pain, it is important to recognise that there was a high level of heterogeneity between the studies in terms of their setting, nationality of population, injury characteristics, mechanism of injury, and assessment time point. However, the attempt to determine estimates of prevalence in a more homogeneous cohort in terms of injury aetiology (ie, combat injury) is in keeping with current suggestions of how to reduce heterogeneity in the exploration of the diverse field of postamputation pain.^66^ The lack of longitudinal data gives us only a single reference point for pain prevalence in these populations and therefore does not describe how pain manifests over time. This is compounded by the fact that, in the majority of studies, minimal information was provided about how the questions around painful symptoms were posed to participants. This means that it is not clear whether point prevalence (pain at the time of the assessment), period prevalence (pain during a specified time period), or lifetime prevalence (pain at any point since injury) was measured in the included studies. This could mean that different types of prevalence are included in one analysis.

Our risk of bias assessment determined that although external validity was high, the majority of studies did not use validated measures for assessing pain and nearly half lacked a robust, specific case definition relating to neuropathic pain or phantom limb pain. This may have led to error in prevalence reported by individual studies, and because we did not exclude studies based on the risk of bias assessment, studies with a potential risk of bias were included in the meta-analysis.

Assimilation of data from the different populations was limited by the lack of clear case definitions and validated evaluation tools. The review also did not assess the impact of trauma related to conflict on civilian populations or in children. In recent conflicts, an increasing number of nonmilitary personnel, such as private contractors and diplomats, were deployed and experienced combat-related injury.^12^ Consequently, the findings may not be generalisable outside of the military or veteran context.

### 4.3. Future research

This systematic review was undertaken in part to inform research into chronic pain in the ADVANCE study, a prospective longitudinal study with matched uninjured controls that aims to evaluate the long-term outcomes of combat trauma in British soldiers injured in Afghanistan between 2002 and 2014.^6^ The outcome measures that we have chosen to use in ADVANCE are designed to provide a standardised assessment of the mechanistic classification, impact, and severity of pain related to combat injury. It will include validated and consensus methods for definitions of neuropathic pain and post amputation pain as well as holistic measures for pain impact.

## 5. Conclusions

This systematic review identifies a prevalence of phantom limb pain of 57% (46%-68%) and residual limb pain of 61% (50%-71%) following combat trauma. Other types of neuropathic pain were less commonly reported. Although there are potentially many aetiological, psychological, treatment, and social differences between military and civilian cohorts that may influence pain prevalence, this prevalence appears similar to rates reported in systematic reviews of civilian or mixed cohorts. The inconsistency in the case definitions, terminology, and measurement of pain impact indicate a need for consensus case definitions and core pain outcome domains in the evaluation of similar cohorts in future research.

## Conflict of interest statement

JV has received consultancy fees from Embody Orthopaedics and Casquar. ASCR undertakes consultancy and advisory board work for Imperial College Consultants; in the last 36 months, this has included remunerated work for Confo, Vertex, Pharmanovo, Lateral, Novartis, Mundipharma, Orion, Shanghai SIMR Biotech, and Asahi Kasei & Toray. ASCR is named as an inventor on patents: Rice A.S.C., Vandevoorde S., and Lambert D.M: Methods using N-(2-propenyl)hexadecanamide and related amides to relieve pain. WO 2005/079,771; Okuse K. et al.: Methods of treating pain by inhibition of vgf activity EP13702262.0/WO2013 110,945. The remaining authors have no conflicts of interest to declare.

## Appendix A. Supplemental digital content

Supplemental digital content associated with this article can be found online at http://links.lww.com/PAIN/B946.
