# Supplementary material for: A systematic review of the prevalence of postamputation and chronic neuropathic pain associated with combat injury in military personnel
Source: Pain. 2023 Dec 15;165(4):727–40. doi: 10.1097/j.pain.0000000000003094 (PMC10949216; doi:10.1097/j.pain.0000000000003094)
Supplement: Supplementary file 2 [file jop-165-727-s002.pdf]

Conflict	First Author	Age at assessment, yrs, average (central tendency)	Male %	Ethnicity %	Service	Serving/Veteran	Relationship status	Rank at time of Injury	Work Status
Iraq and Afghanistan	Aldington	28.8 (6.7)	NR	NR	NR	Serving	NR	NR	NR
	Bedigrew	23.4 (19-36)	NR	NR	NR	NR	NR	NR	NR
	Birch	26.5 (18.1 - 42.6)	98%		NR	Serving	NR	NR	NR
	Buchheit	26.9	98%	88.6 % White<br>6% African American<br>2.5% American Indian<br>1.3% Hawaiian,<br>1.3% Asian	NR	Serving	NR	NR	NR
	Duffy	26 (24-31)	NR	NR	Soldiers	NR	66% married	NR	94.3% employed
	Ketz	25.3	100%	NR	NR	NR	NR	NR	NR
	Krueger	26 (20-42)	NR	NR	NR	NR	NR	NR	NR
	Rafferty	26.3 (5.2)	98%	NR	NR	NR	NR	NR	NR
	Rauh	<25= 55%<br>25-29= 24% >30= 20%	98%	NR	71% Army<br>1.4% Air Force<br>24% Marine Corps<br>3.4% Navy	59% Veteran, 41% Serving			
	Rieber (OIF/OEF)	29.3 Years	96.8%	73% Caucasian<br>8.9% Black<br>5% American Indian<br>1.4% Asian<br>9.6% Hispanic<br>0.7% Hawaiian	NR	20.5% Active duty	60.6% Married	11% Officers, 89% Enlisted	53.6% Employed, 22.6% Students

	Rivera	25 (18-49)	88%	NR	NR	NR	NR	NR	NR
	Tintle	26 (22-30)	NR	NR	NR	NR	NR	NR	NR
Iran-Iraq	Allami	52.4 (7.4)	99.2%	NR	NR	Veteran	97.2% Married	NR	34% employed
	Ebrahimzadeh (2006)	41.6	NR	NR	Soldiers	Veterans	96% Married	NR	60% employed
	Ebrahimzadeh (2007)	NR	100%	NR	Combatants	Veterans	100% Married	NR	48.1% employed
	Ebrahimzadeh (2009a)	43.3 (10.4)	100%	NR	Soldiers	Veterans	91% married	NR	62% employed
	Ebrahimzadeh (2009b)	NR	NR	NR	Soldiers	Veterans	100% married	NR	65% employed
	Ebrahimzadeh (2013)	44.1 (7)	NR	NR	Soldiers	Veterans	93.4% married	NR	NR
	Ebrahimzadeh (2016)	45 (37-89)	97%	NR	Combatants	Veterans	96% married	NR	77% employed
	Esfandiari	43 (6.48)	100%	NR	Combatants	Veterans	NR	NR	27% employed
	Faraji	51.5 (5.5)	100%	NR	Combatants	Veterans	99% married	NR	NR
	Rayegani	<25= 1.4%<br>25-34= 3.8%<br>35-44= 65.3%<br>45-54= 25.6%<br>>55= 3.5%	97.6%	NR	NR	NR	97.6% married	NR	73.1% employed<br>2.7% student
	Taghipour	42.5 (36-63)	100%	NR	Combatants	Veterans	100%	NR	60%
Vietnam	Foote	NR	100%	NR	Soldiers	Veterans	NR	NR	NR
	Reiber (Vietnam Cohort)	60.7	100%	81% Caucasian<br>6.8% Black<br>1% American Indian		Veteran	74.8% Married	7% Officers, 93% Enlisted	78.7% Employed

				0.7% Asian,<br>9.1% Hispanic<br>0.7% Hawaiian					
	Rothberg	22.2	NR	NR	NR	NR	NR	NR	NR
Other/Mixed conflicts	Gunawardena	30.2 (SD4.6)	100%	NR	Army	Actively serving and veterans<br>(319 employed by army)	80.5% Married		69.2% employed in the Army
	Sherman (1983)	NR	NR	NR	NR	NR	NR	NR	NR
	Sherman (1984)	51.4 (13.4) PLP;<br>52.7(13.1) No PLP	NR	NR	NR	NR	NR	NR	NR
	Sherman (1999)	32	95.50%	NR	NR	NR	NR	NR	44%
	Rathore	11-20= 8.1%, 21-30= 57.7%, 31-40= 28.5%, 41-50= 5.7%	100%	NR	Army	NR	NR	NR	NR
	Wartan	73	NR	NR	NR	Veterans	NR	NR	NR

Supplemental Table 1: Participant characteristics

NR=Not reported; FT=Full time; PT=Part time; PLP= Phantom Limb Pain

Conflict	First Author	Age at time of injury. Average (central tendency)	Time since injury (weeks), mean	Blast (%)	Penetrating (%)	Blunt (%)	NISS, mean	Amputation Traumatic (%)	Amputation Delayed (%)	Upper Limb Amputations, n	Lower Limb Amputations, n	TBI %
Iraq and Afghanistan	Aldington	NR	<13= 15<br>13-26=6<br>26-52=11<br>>52=16	NR	NR	NR	NR	100%	NR	11	54	NR
	Bedigrew	NR	74	84	5	2	NR		NR	NR	NR	NR
	Birch	NR	82	63	37	NR	18.4	18	NR	11	113	NR
	Buchheit	NR	35.6	NR		NR	NR	100	NR	NR	NR	NR
	Duffy	NR	NR	87	13	NR	60.4% >16	NR	NR	NR	NR	NR
	Ketz	NR	499.2	NR	NR	NR	NR	41	38	7	23	NR
	Krueger	NR	82.2	70	24	2	14.8	NR	100	NR	NR	NR
	Rafferty	NR	52.8	NR	NR	NR	NR	NR	NR	7	106	NR
	Rauh	NR	NR	87.4	NR	NR	54% 1-15, 45% >16	NR	NR	115	415	33.3
	Rieber (OIF/OEF)	NR	166	NR	NR	NR	NR	NR	NR	NR	NR	33.9
	Rivera	NR	25	77	23	NR	11.2	NR	NR	NR	NR	NR
	Tintle	NR	20.1	87	8	NR	13	NR	3	100	18	NR
Iran-Iraq	Allami	NR	1547.5	63.9	27.1	NR	NR	100	NR	NR	247	NR
	Ebrahimzadeh (2006)	23.06	910	48	52	NR	NR	40	60	25	NR	NR
	Ebrahimzadeh (2007)	21.29 (16-54)	910	66	33.3	NR	NR	63	37	NR	27	NR
	Ebrahimzadeh (2009a)	25.5 (10.9)	902.7	45.1	54.8	NR	NR	Mixed	Mixed	NR	31	NR
	Ebrahimzadeh (2009b)	23.1 (14-60)	904.8	68	30	3	NR	59	41	NR	96	NR
	Ebrahimzadeh (2013)	NR	1383.2	NR	NR	NR	NR	60.5	39.5	NR	76	NR
	Ebrahimzadeh (2016)	20 (14-65)	1320.8	43	50	NR	NR	63	37	15	291	NR

	Esfandiari	21.5 (6.1)	1144	NR	NR	NR	NR	NR	NR	NR	587	NR
	Faraji	NR	1647.4	NR	80	NR	NR	96	4	3	NR	NR
	Rayegani	NR	NR	NR	NR	NR	NR	NR	NR	0	670	NR
	Taghipour	23.3 (16.4)	1123.2	61	15.6	NR	NR	50.	49.6	0	141	NR
Vietnam	Foote	NR	NR	NR	NR	NR	NR	NR	NR	91	231	NR
	Reiber (Vietnam)	NR	NR	NR	NR	NR	NR	NR	NR	NR	NR	3.4
	Rothberg	NR	NR	NR	NR	NR	NR	NR	NR	NR	NR	NR
Other/Mixed conflicts	Gunawardena	26.2(4.5)	NR	86.6%	NR	NR	NR	NR	NR	0	461	NR
	Sherman (1983)	24.9 (5.6)	1503 PLP<br>1336 No PLP	NR	NR	NR	NR	NR	NR	137	657	NR
	Sherman (1984)	NR	436	NR	NR	NR	NR	NR	NR	11	51	NR
	Sherman (1999)	25.7 (9.1)	1456	NR	NR	NR	NR	NR	NR	NR	NR	NR
	Rathore	NR	NR	59.7	8.9	10.6	NR	NR	NR	NR	130	NR
	Wartan	25.8 PLP<br>25.2 No PLP	2600	NR	NR	NR	NR	NR	NR	99	471	NR

Supplemental Table 2: Injury characteristics

TBI=Traumatic Brain Injury; NR= Not reported; NISS= New Injury Severity Score; PLP= Phantom Limb Pain

Conflict	First Author	Back pain prevalence (%)	Other pain sites reported (%)	PTSD Tool(Cutoff)	PTSD Prevalence (%)	Depression Tool(Cutoff)	Depression Prevalence (%)	Anxiety Prevalence (%)	QoL measure used	Reported QoL (% or mean(SD))
Iraq and Afghanistan	Aldington									
	Bedigrew									
	Birch									
	Buchheit			PCL-M(>50)		PHQ-9(>10)				
	Duffy			PCL-Civ (>44)	13.2%	HAD (>8)	11.3	18.9	EuroQoL VAS	Non-NeuP = 90 (75–92) Possible NeuP = 70 (65–85); Definite NeuP = 80 (65–85)
	Ketz						10			
	Krueger				31					
	Rafferty									
	Rauh									
	Rieber (OIF/OEF Cohort)	42.1 LBP	21.9 Migraine		58.7		24.0		Single-item global measure of quality of life*	85.5% Good, very good or excellent
	Rivera		24% Painful scar							
	Tintle									
Iran-Iraq	Allami									
	Ebrahimzadeh (2006)									
	Ebrahimzadeh (2007)	44.4 LBP	33.3 Contralateral knee 14.8 Ipsilateral knee							
	Ebrahimzadeh (2009a)	61.2 LBP	54.8 Chronic contralateral knee 14.7 Ipsilateral hip		32		9.6	16.1		

	Ebrahimzadeh (2009b)	44 LBP	38 Contralateral knee,<br>13 Ipsilateral knee		32		22	22		
	Ebrahimzadeh (2013)	44.7 Cervical<br>31.6 Thoracic<br>72.5 Lumbar<br>17.1 Sacral	80.3 in upper extremities						SF-36	PCS 45.9(21.6)<br>MCS 58.0 (25.2)
	Ebrahimzadeh (2016)									
	Esfandiari	69 LBP	67 Contralateral joint							
	Faraji	80 LBP						54.5	SF-36	PCS 33.3 (22.0)<br>MCS 48.6 (29.6)
	Rayegani	53 LBP<br>9 thoracic<br>22 neck								
	Taghipour	76.6 LBP	79.4 Contralateral leg articular						SF-36	PCS 43.0 (15.9)<br>MCS 52.7 (16.8)
Vietman	Foote	76.5 LBP		PCL-M (>50)	15.80	PHQ-9 (>10)	17.80		Modified SF-12	PCS 36.4 (10.5)<br>MCS 47.2 (7.7)
	Reiber (Vietnam Cohort)	36.2 LBP	11.4 Migraine		37.60		24.50		Single-item global measure of quality of life*	70.7% Good, very good or excellent
	Rothberg									
Other/Mixed conflicts	Gunawardena								SF-36	PCS 60.7 (10.4),<br>MCS 67.9 (10.9)
	Sherman 1983									
	Sherman (1984)									

	Sherman (1999)								Self-report of own health status	16/45 Health as good as it ever was, 19/45 good, 9/45 fair, 1/45 poor
	Rathore									
	Wartan									

Supplemental Table 3: Psychological and Quality of Life aspects reported in the included studies.
**data extracted from associated publication addressing the same cohort and referenced in the original manuscript (Epstein et al. Quality of life for veterans and servicemembers with major traumatic limb loss from Vietnam and OIF/OEF conflicts. JRRD 2010.; 47:373-386). NR=Not recorded; OEF=Operation Enduring Freedom; OIF=Operation Iraqi Freedom; PTSD=Post Traumatic Stress Disorder; LBP=Low Back Pain; PCL-M=Posttraumatic Stress Disorder Checklist – Military; PCL-Civ=Posttraumatic Stress Disorder Checklist – Civilian; PHQ-9=Patient Health Questionnaire -9; SF-36=Short Form 36 Health Survey; SF-12=Short Form 12 Health Survey; PCS=Physical Component Score; MCS=Mental Component Score*

Appendix 2

1. Veterans/
2. Veteran* mp
3. Veterans health/
4. veteran* adj3 health
5. Military medicine mp
6. Military medicine/
7. Hospitals, Veterans/
8. veteran* adj3 hospital*
9. military adj3 person*
10. military personnel/
11. ex-military mp
12. ex-service mp
13. ex-forces mp
14. active* adj3 servi*
15. army mp
16. armed forces mp
17. soldier/
18. soldier* mp
19. 1 or 2 or 3 or 4 or 5 or 6 or 6 or 8 or 9 or 10 or 11 or 12 or 13 or 14 or 15 or 16 or 17 or 18
20. Wounds, gunshot/
21. gun shot mp
22. gunshot mp
23. blast adj3 injur* mp
24. blast injuries/
25. land mine* mp
26. shell injur*
27. explosion*
28. bomb adj3 injur*
29. battle adj3 injur*
30. war exposure/
31. Warfare mp
32. warfare/
33. war/
34. war related injuries/
35. war adj3 injur* mp
36. conflict/
37. combat adj3 injur*mp
38. combat adj3 wound* mp
39. combat adj3 trauma* mp
40. wounds and injuries/
41. wound* mp
42. trauma mp
43. amputation/

44. amputation* mp
45. amputation, traumatic/
46. trauma* adj3 amputation mp
47. amputation, stumps/
48. amputation* adj3 stump* mp
49. limb* adj3 loss* mp
50. Traum* adj3 brain injury
51. mTBI mp
52. Brain Concussion/
53. Brain concussion mp
54. Post concussion syndrome/
55. Post concussion syndrome mp
56. Spinal injur*
57. Leg injur*
58. Limb injur*
59. Peripheral nerve injuries/
60. Peripheral nerve injur* mp
61. 20 or 21 or 22 or 23 or 24 or 25 or 26 or 27 or 28 or 29 or 30 or 31 or 32 or 33 or 34 or 35 or 36 or 37 or 38 or 39 or 40 or 41 or 42, 43, 44, 45, 46, 47, 48 or 49 or 50 or 51 or 52 or 5 or 54 or 55 or 56 or 58 or 58
62. neuropathic pain* mp
63. chronic adj3 neuropathic pain* mp
64. exp Neuralgia/
65. Phantom limb/
66. Phantom limb* mp
67. Phantom pain/
68. Phantom pain* mp
69. Phantom sensation* mp
70. Phantom adj3 pain mp
71. Complex regional pain syndromes/
72. Complex regional pain syndrome* mp
73. CRPS mp
74. 62 or 63 or 64 or 65 or 66 or 67 or 68 or 69 or 70 or 71 or 72 or 73
75. 19 and 61 and 74

Appendix 3

Title	Author	Year	DOI	Exclusion Reason
Health-related quality of life and the ability to perform activities of daily living: a cross-sectional study on 1079 war veterans with ankle-foot disorders	Allam	2017	10.1186/s40779-017-0146-1	Wrong outcomes
Bilateral lower limb amputations as a result of landmine injuries	Atesalp	1999	10.3109/03093649909071610	Wrong patient population
Frequency and Severity of Phantom Limb Pain in Veterans with Major Upper Limb Amputation: Results of a National Survey	Balakhanlou	2021	https://doi.org/10.1002/pmrj.12485	Mixed population
Chronic posttraumatic stress disorder and chronic pain in Vietnam combat veterans	Beckham	1997	10.1016/s0022-3999(97)00129-3	Wrong outcomes
Fate of combat nerve injury	Beltran	2012	10.1097/BOT.0b013e31823f000e	Wrong outcomes
Pain following battlefield injury and evacuation: a survey of 110 casualties from the wars in Iraq and Afghanistan	Buckenmaier	2009	10.1111/j.1526-4637.2009.00731.x	Wrong patient population
Survey of Phantom Limb Pain, Phantom Sensation and Stump Pain in Cambodian and New Zealand Amputees	Byrne	2011	10.1111/j.1526-4637.2011.01105.x	Mixed population
Prevalence and characteristics of phantom limb pain and residual limb pain in the long term after upper limb amputation	Desmond	2010	10.1097/MRR.0b013e328336388d	Mixed population
Phantom Pain and Risk Factors: A Multivariate Analysis	Dijkstra	2002	https://doi.org/10.1016/S0885-3924(02)00538-9	Wrong patient population
Persian Gulf War amputees: injuries and rehabilitative needs	Dillingham	1994		Wrong outcomes
Long-term follow-up study of bilateral above-the-knee amputees from the Vietnam War	Dougherty	1999	10.2106/00004623-199910000-00003	Wrong outcomes
Transtibial amputees from the Vietnam War. Twenty-eight-year follow-up	Dougherty	2001	10.2106/00004623-200103000-00010	Wrong outcomes
Long-term follow-up of unilateral transfemoral amputees from the Vietnam war	Dougherty	2003	10.1097/01.Ta.0000046260.16866.A9	Wrong outcomes
Long-term follow-up of unilateral above-knee amputees	Dougherty	2000		Wrong outcomes
The Military Extremity Trauma Amputation/Limb Salvage (METALS) study: outcomes of amputation versus limb salvage following major lower-extremity trauma	Doukas	2013	10.2106/jbjs.K.00734	Wrong outcomes
Chronic phantom sensations, phantom pain, residual limb pain, and other regional pain after lower limb amputation	Ehde	2000	10.1053/apmr.2000.7583	Wrong patient population
Phantom Pain, Residual Limb Pain, and Back Pain in Amputees: Results of a National Survey	Ephraim	2005	https://doi.org/10.1016/j.apmr.2005.03.031	Wrong patient population

Unilateral lower-limb loss: prosthetic device use and functional outcomes in servicemembers from Vietnam war and OIF/OEF conflicts	Gailey	2010	10.1682/jrrd.2009.04.0039	Wrong outcomes
Phantom limb pain and residual limb pain following lower limb amputation: a descriptive analysis	Gallagher	2001	10.1080/09638280010029859	Wrong patient population
Chronic pain associated with upper-limb loss	Hanley	2009	10.1097/PHM.0b013e3181b306ec	Wrong patient population
Sympathectomy for causalgia: experience with military injuries	Hassantash	2000	10.1097/00005373-200008000-00012	Wrong outcomes
Risk factors for and results of late or delayed amputation following combat-related extremity injuries	Helgeson	2010	10.3928/01477447-20100722-02	Wrong outcomes
Phantom limbs	Henderson	1948	10.1136/jnnp.11.2.88	Wrong study design
Evaluation of problems and needs of veteran lower-limb amputees in the San Francisco Bay Area during the period 1977-1980	Hoaglund	1983		Wrong outcomes
Phantom pain and phantom sensations in upper limb amputees: an epidemiological study	Kooijman	2000	10.1016/s0304-3959(00)00264-5	Wrong patient population
Initial injury severity and social factors determine ability to deploy after combat-related amputation	Krueger	2014	https://doi.org/10.1016/j.injury.2014.02.008	Duplicate
Chronic low back pain in traumatic lower limb amputees	Kulkarni	2005	10.1191/0269215505cr819oa	Wrong patient population
Pain in traumatic upper limb amputees in Sierra Leone	Lacoux	2002	10.1016/s0304-3959(02)00154-9	Wrong patient population
Pain in traumatic upper limb amputees in Sierra Leone	Lacoux	2002	10.1016/s0304-3959(02)00154-9	Wrong patient population
Prevalence of chronic pain, posttraumatic stress disorder, and persistent postconcussive symptoms in OIF/OEF veterans: polytrauma clinical triad	Lew	2009	10.1682/jrrd.2009.01.0006	Wrong outcomes
Functional outcomes following lower limb amputation at the armed forces institute of rehabilitation medicine using lower extremity functional scale	Mansoor	2013		Wrong outcomes
Resource utilization and disability outcome assessment of combat casualties from Operation Iraqi Freedom and Operation Enduring Freedom	Masini	2009	10.1097/BOT.0b013e31819dfa04	Wrong outcomes
A comparison of health outcomes for combat amputee and limb salvage patients injured in Iraq and Afghanistan wars	Melcer	2013	10.1097/TA.0b013e318299d95e	Mixed population
Short-Term Physical and Mental Health Outcomes for Combat Amputee and Nonamputee Extremity Injury Patients	Melcer	2013	10.1097/BOT.0b013e3182517e1c	Wrong outcomes
A Comparison of Four-Year Health Outcomes following Combat Amputation and Limb Salvage	Melcer	2017	10.1371/journal.pone.0170569	Wrong outcomes

Phantom pain in bilateral upper limb amputation	Modirian	2009	10.1080/09638280902810976	Wrong patient population
A clinical evaluation of stumps in lower limb amputees	Pohjolainen	1991	10.3109/03093649109164285	Wrong patient population
Pain and health-related quality of life in war veterans with bilateral lower limb amputations	Rahimi	2012	10.5812/traumamon.5135	Wrong patient population
Outcomes of IED foot and ankle blast injuries	Ramasamy	2013	10.2106/jbjs.K.01666	Wrong outcomes
Initial treatment of combat related limb injuries in Colombia	Rondanelli	2016	10.18273/revsal.v48n3-2016003	Wrong outcomes
Phantom limb, residual limb, and back pain after lower extremity amputations	Smith	1999	10.1097/00003086-199904000-00005	Wrong patient population
Phantom Pain and Health-Related Quality of Life in Lower Limb Amputees	vanderSchans	2002	https://doi.org/10.1016/S0885-3924(02)00511-0	Wrong patient population
Fate of the retained lower limb joints in Second World War Amputees	Hungerford	1975		Wrong outcomes
The influence of phantom limb	Feinstein	1954		Wrong outcomes
